# A Novel Algorithm for the Assessment of Blood-Brain Barrier Permeability Suggests That Brain Topical Application of Endothelin-1 Does Not Cause Early Opening of the Barrier in Rats

**DOI:** 10.1155/2011/169580

**Published:** 2011-03-30

**Authors:** D. Jorks, D. Milakara, M. Alam, E. J. Kang, S. Major, A. Friedman, J. P. Dreier

**Affiliations:** ^1^Department of Experimental Neurology, Charité University Medicine Berlin, 10098 Berlin, Germany; ^2^Center for Stroke Research Berlin, Charité University Medicine Berlin, Charitéplatz 1, 10117 Berlin, Germany; ^3^Bernstein Center for Computational Neuroscience Berlin, 10115 Berlin, Germany; ^4^Department of Neuroradiology, Charité University Medicine Berlin, 10098 Berlin, Germany; ^5^Department of Neurosurgery, Hannover Medical School, 30625 Hannover, Germany; ^6^Department of Neurology, Charité University Medicine Berlin, 10098 Berlin, Germany; ^7^Departments of Physiology, Neurosurgery, and Biomedical Engineering, Ben-Gurion University of the Negev, Beersheva 84105, Israel

## Abstract

There are a number of different experimental methods for *ex vivo* assessment of blood-brain barrier (BBB) opening based on Evans blue dye extravasation. However, these methods require many different steps to prepare the brain and need special equipment for quantification. We here report a novel, simple, and fast semiquantitative algorithm to assess BBB integrity *ex vivo*. The method is particularly suitable for cranial window experiments, since it keeps the spatial information about where the BBB opened. We validated the algorithm using sham controls and the established model of brain topical application of the bile salt dehydrocholate for early BBB disruption. We then studied spreading depolarizations in the presence and the absence of the vasoconstrictor endothelin-1 and found no evidence of early BBB opening (three-hour time window). The algorithm can be used, for example, to assess BBB permeability *ex vivo* in combination with dynamic *in vivo* studies of BBB opening.

## 1. Introduction

Blood-brain barrier (BBB) disruption has been investigated in a multitude of experimental studies on cerebral pathologies. These include ischemic stroke, epilepsy, spreading depolarizations (SD) in otherwise healthy naïve tissue [1–4], or effects of detergents such as bile salts [5]. However, the knowledge on the early time course of BBB disruption is limited in these conditions. It is of particular interest for future clinical prevention and treatment of brain disorders associated with BBB impairment to characterize the early phase of BBB opening for the following reasons.

(i) From that moment on, damage of brain tissue can aggravate due to blood components that can now enter the brain without restriction.

(ii) Secondary effects associated with BBB opening such as edema and cerebral hemorrhage may limit the application of early therapies to a certain time window. This, for example, seems to apply to systemic thrombolysis with recombinant tissue plasminogen activator (rt-PA) in ischemic stroke which is limited to the 4.5-hour time window.

(iii) The onset of disruption marks the time point when drugs that are normally kept from entering the brain become able to cross the barrier. This may be relevant for neuroprotective treatment that has been shown to be most effective when given either before or early after an initial insult. If a given neuroprotectant does not normally pass through the barrier but only crosses the barrier after disruption, it is important to determine whether BBB opening occurs early enough for the neuroprotectant to have a sufficient effect on its target in the parenchyma yet.

Even less is known about early BBB opening in gradually developing focal ischemia since most animal models of focal ischemia are designed to replicate severe sudden onset ischemic events in humans occurring after embolic or thrombotic occlusion of a large vessel. Gradually developing focal ischemia, however, is of similar importance for human stroke. It may, for example, occur in patients with delayed cerebral ischemia after aneurismal subarachnoid hemorrhage or in vasculitides, and so forth.

Here, we compared early BBB opening in rats at three hours in (i) the brain topical endothelin-1 (ET-1) model of focal cerebral ischemia, (ii) SDs propagating through healthy naïve cortex, (iii) the topical bile salt dehydrocholate (DHC) model, and (iv) a sham control. For this purpose we developed a simple, semiquantitative algorithm to assess BBB disruption using the target to background ratio. We chose the brain topical ET-1 model, since it allowed us to induce a gradually developing focal ischemia by titrating the constrictive effect of different concentrations of ET-1 on the cerebral vasculature. This results in features such as the absence of a terminal SD, clearly different to the classical and widely distributed model of middle cerebral artery occlusion. Instead, the brain topical ET-1 model is characterized by clusters of recurrent, prolonged SDs that ride on an ultraslow negative potential shift and lead to a persistent depression of brain electrical activity [6, 7]. Despite the absence of terminal SD, histological assessment indicated previously neuronal death in the cortex exposed to ET-1 [8]. Interestingly, we did not find evidence of early BBB disruption in cortex exposed to ET-1 in the present study in contrast to cortex exposed to DHC that served as a positive control.

## 2. Materials and Methods

### 2.1. Animals

Male Wistar rats (*n* = 28; 250–350 g) were anaesthetised with 100 mg/kg body weight thiopental-sodium (Trapanal, BYK Pharmaceuticals, Konstanz, Germany) intraperitoneally, tracheotomised, and artificially ventilated (Effenberger Rodent Respirator, Effenberger Med.-Techn. Gerätebau, Pfaffing/Attel, Germany).

After cannulation of the left femoral artery and vein both vessels were continuously infused with saline solution at 1 mL/h. Body temperature was maintained at 38.0 ± 0.5°C using a heating pad. At all times during the experiments, mean arterial pressure (MAP; RFT Biomonitor, Zwönitz, Germany) and end-expiratory partial pressure of carbon dioxide (Heyer CO_2_ Monitor EGM I, Bad Ems, Germany) were monitored, whereas arterial partial pressure of oxygen (paO_2_), carbon dioxide (paCO_2_), and pH were measured serially using a Compact 1 blood gas analyser (AVL Medizintechnik GmbH, Bad Homburg, Germany).

An open cranial window was implanted as reported previously [9–11]. First, a parietal craniotomy of 7 × 4 mm was performed using a saline-cooled drill. Then, wax and dental cement (Paladur) were used to build the outer rim of the cranial window. After removal of the dura mater, an inflow tube was inserted in the wax rim to later superfuse the brain cortex with artificial cerebrospinal fluid (aCSF). ACSF containing (mmol/L) K^+^ 3, Na^+^ 152, Ca^2+^ 1.5, Mg^2+^ 1.2, HCO_3_^−^ 24.5, Cl^−^ 135, glucose 3.7, and urea 6.7 was equilibrated with a special gas mixture in order to yield physiological pH and partial gas pressures. A second small drill hole in the temporal bone served to later elicit SD by subdural application of potassium chloride (KCl, 150 mmol/L).

Two glass microelectrodes were positioned at the window site in a cortical depth of 300 *μ*m below surface to record the intracortical electrocorticogram (ECoG). Alternate current (AC)-ECoG (bandpass: 0.5–45 Hz) and direct current (DC)-ECoG of each electrode were obtained using a differential amplifier (Jens Meyer, Munich, Germany).

### 2.2. Experimental Protocols

After surgery, the recording was started, and the cranial window was first superfused with aCSF for one to one and a half hours to obtain baseline parameters. In all four groups, 1 mL of 2% Evans blue was then slowly administered intravenously for five minutes.

Shortly thereafter, DHC (2 mmol/L in aCSF) was applied brain topically in the DHC group (group 1, *n* = 8), while in the sham-operated control group (group 2, *n* = 6), physiological aCSF was applied continuously for the whole period of measurement. In animals of the ET-1 (Sigma-Aldrich inc., Steinheim, Germany) group (group 3, *n* = 7), ET-1 was administered brain topically in stepwise increases from 10^−8^ to 10^−7^ to 10^−6^ mmol/L at one-hour intervals as described previously [11]. In group 4 (*n* = 5), physiological aCSF was applied brain topically in the recording window, while SDs were induced manually with a droplet of KCl in a remote window at three different time points. From the remote window, the SDs propagated to the naïve recording window equipped with the microelectrodes.

**Figure 1:**
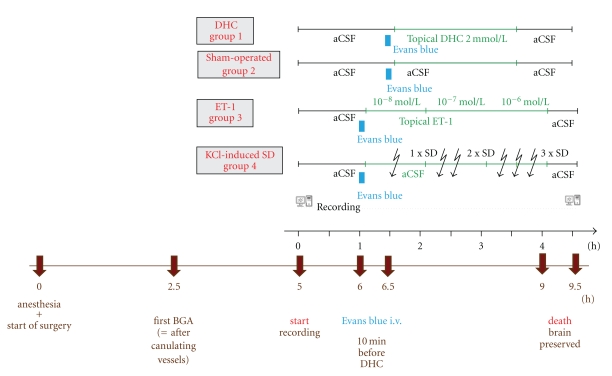
Experimental protocols. For a better overview, the important time points of the experimental protocols are visualized here. The black time line shows the recording period starting at the end of surgery. The brown time line shows the overall experimental time starting from anesthesia until death of the animals. BGA = blood gas analysis.

Three hours after the potentially BBB compromising events and approximately seven hours after the start of the head surgery (Figure 1), rats of all groups were sacrificed by decapitation. Brains were then extracted, rinsed carefully with saline solution, and preserved in cold 4% paraformaldehyde (PFA) solution for at least 48 hours. Each brain was later cut into five coronal slices of 2 mm thickness at and around the window area. Pictures of all slices were taken at 96 dots per inch (dpi) resolution (1040 × 1392 pixel) as 48-bit RGB images (16 bits per channel) using a digital microscope camera (magnification ×10) (Leica DFC300 FX Digital Color Camera, Leica Microsystems AG, CH-9435, Heerbrugg, Switzerland).

### 2.3. Image Processing

In brief, brain slice images were imported as Matlab variables: three-dimensional matrices with the third dimension containing the three color components of the RGB color model, while the other two dimensions represent width and length of the image. In the algorithm, the blue channel is selected from the RGB image which renders the variables two-dimensional for further calculations. Both, the three- and two-dimensional variables are stored in the unsigned integer 16 bit (uint16) format. The minimum intensity of each image was arbitrarily set to zero and the maximum intensity to 2^16^, the maximum value of the uint16 format. The term filter used in the algorithm does not refer to technical but image filters. Those are selecting mechanisms depending on intensities and gradients (first derivatives of intensities) or using the “canny method” for edge detection succeeded by contour interpolation. Thresholds for the image filters were adjusted manually depending on the image quality. The filters were used to distinguish brain from background of the image. Pixel values were set to “one” when representing brain and to “zero” when representing background. The information of the thus-generated binary mask stores the location of the brain. This spatial information can easily be accessed later by matrix multiplication. The target to background ratio (TBR) was calculated by dividing the median intensity of the window area (target) by the corresponding value of the contralateral hemisphere (background). Both intensity values were normalized to an ipsilateral remote area. The algorithm is further explained in Section 3.

### 2.4. Data Analysis

Data were analyzed by comparing absolute changes of the DC-ECoG potential and relative changes in Evans blue dye extravasation. Statistical tests are mentioned more specifically in the results section. A *P*-value of <.05 was considered statistically significant. Data in text and figures are given as median value as well as first and third quartiles in parentheses.

## 3. Results

### 3.1. Evaluation Method of Evans Blue Dye Extravasation

BBB disruption was assessed by calculating the TBR of relative Evans blue dye extravasation in the cranial window region compared to the corresponding region of the contralateral hemisphere. 

**Figure 2:**
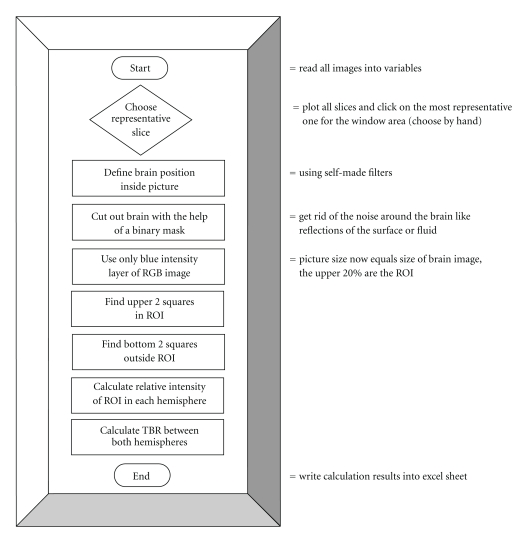
Algorithm.

**Figure 3:**
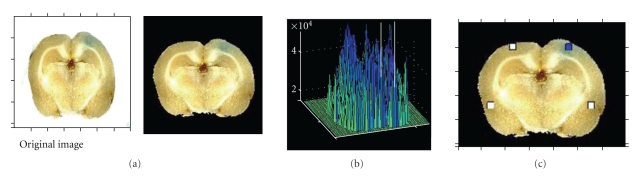
Steps of the algorithm. This figure shows how an original picture, taken by a digital camera, is processed using the Matlab routine. (a) First, the brain was identified within the picture, and a mask was created to reduce the background noise. (b) Then, the blue layer of the picture was analyzed, and the intensity scale was adjusted. (c) The four squares were chosen, and the target to background ratio was calculated.

The digital images of all slices (five per brain) were imported into Matlab variables and screened manually for one representative slice of each rat brain that contained the region of the cranial window. The chosen slices were then further processed using the custom-made Matlab routine as described (see also Figure 2 for the algorithm used). The location of the brain was identified within the picture by filters and displayed through a binary mask. The image was then cropped (cutout image) down to the outermost “brain pixel” identified by the filter process. The upper 20% (value chosen manually) of the cutout brain image were defined as the region of interest (ROI) that approximately represented the area between the upper cortical surface and the ventricles. The routine provided a 50 × 50 pixel square (~0.3 mm^2^, blue square in Figure 3(c)) within the ROI of the ipsilateral hemisphere (hemisphere with the cranial window) and a corresponding equally sized square (white square in Figure 3(c)) within the ROI of the contralateral hemisphere. Furthermore, the algorithm delivered two corresponding equally sized squares in the bottom region of the image (one bottom square in each hemisphere), clearly outside the ROI that were used to normalize the median intensities to yield a relative value for each side. The size of the four squares (50 by 50 pixels) was chosen according to the width of the cranial window (~4 mm) and thickness of the rat cortex. The initial location of the first square was user-selected according to the center of the cranial window area. This position was stored using distance values from the edges of the mask.

Since RGB is an additive color model, the blue channel of the images represents best the amount of blue dye (Evans blue). Therefore, the relative median blue intensities of both, the ipsilateral (blue square in the upper right hemisphere of Figure 3(c) = target) and the contralateral side (corresponding white square in the upper left hemisphere of Figure 3(c) = background) were used to calculate the TBR. As a visual control the routine plotted all four squares (blue and white ones) found in the image (Figure 3(c)).

### 3.2. DHC Induced Early BBB Opening Was Detected by the Novel Algorithm

The systemic variables measured in this study were mean arterial pressure, arterial pCO_2_, arterial pO_2_, and pH. Mean arterial pressure above 75 mmHg, arterial pO_2_ between 90 and 130 mmHg, arterial pCO_2_ between 35 and 45 mmHg, and arterial pH between 7.35 and 7.45 were accepted as being physiological [8]. Those systemic variables remained within normal limits throughout the preparation and experiments in the four experimental groups.

We first evaluated the brains of eight DHC-treated (group 1) and six sham-operated rats (group 2), since it was shown previously that topical application of DHC is a robust method for early BBB disruption [5]. These two groups served as positive and negative controls for BBB opening, respectively, in order to determine whether our fast algorithm would be able to detect the leakage of Evans blue into the brain tissue.

**Figure 4:**
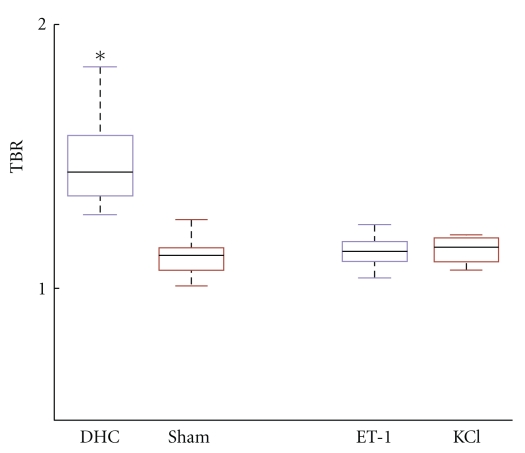
BBB opening. DHC-treated cortex shows significant (*) Evans blue leakage in contrast to the three other groups (Kruskal-Wallis ANOVA on ranks test with Dunn's post hoc analysis, *P* < .05).

Brain topical application of DHC led to a significant increase in Evans blue extravasations compared to the sham operated group (Kruskal-Wallis ANOVA on ranks test with Dunn's post hoc analysis, *P* < .05, Figure 4). DHC did not induce SDs. Neither brain topical application of ET-1 (group 3, *n* = 7) nor SDs propagating through the naïve window area (group 4, *n* = 5) led to any changes in Evans blue extravasations compared to the sham-operated animals at this early time point (Figure 4).

**Figure 5:**
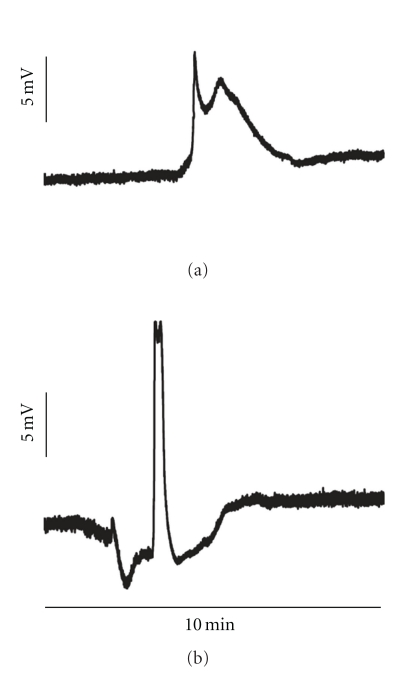
SDs in presence and absence of ET-1. (a) shows a typical SD in the cortex exposed to ET-1, while (b) demonstrates a typical SD in naïve cortex. Note the difference in duration and shape of the SDs. It has been suggested that ET-1 induces conditions of an ischemic penumbra. In the ischemic penumbra, SDs are typically prolonged.

In group 3, topical application of ET-1 induced 4 (2–6) SDs with an amplitude of −13.6 (−18.2–(−11.9)) mV and a duration of 104 (71–215) s, while, in group 4, 7 (7–9) SDs occurred with an amplitude of −13.7 (−16.8–(−9.2)) mV and a duration of 46 (35–50) s. The duration of SDs was significantly longer in presence of ET-1 (*P* = .018, Mann-Whitney rank sum test) [6] (Figure 5).

## 4. Discussion

We developed a novel, simple, and fast algorithm to assess BBB disruption *ex vivo*. Using this algorithm, we confirmed previous findings that brain topical application of the bile salt DHC causes early BBB opening. Neither ET-1-induced SDs nor SDs propagating in naïve cortex led to early BBB opening within the three-hour time window.

Our *ex vivo* algorithm is based on Evans blue dye extravasations. Evans blue, discovered in 1885 by Paul Ehrlich [12], binds to albumin with high affinity. This large complex of serum protein and vital dye cannot cross the intact BBB. Methods already exist to assess *ex vivo* Evans blue leakage from the cerebral vasculature into the brain tissue. However, these methods include several time-consuming steps to prepare the animal brain, and they also require special equipment for quantification. Therefore, they are more costly and time-consuming. This applies to the commonly used method of Katayama and colleagues [13], for example, which is outlined briefly in [4]. At first, Evans blue is injected intravenously in a 2% solution in saline (3 mL/kg body weight) either before or shortly after an insult. Perfusion with physiological saline of the deeply anesthetized animal is necessary to remove the dye inside the vessels at the end of the experiment. After sacrificing the animal and extraction of the brain, either whole hemispheres or brain samples from each side are chosen, weighed, and soaked overnight in potassium hydroxide at 37°C. The alkaline solution is then neutralized by adding phosphoric acid and acetone and shaken vigorously. Thereafter, the solution is centrifuged three times at 3500 rpm for 15 minutes and the absorbance of the extracted solution is measured using a spectrophotometer. Quantitative calculation of the dye content in the brain is then performed based on external standards.

In our current study, we aimed at an easier, less time- and resources-consuming, semiquantitative *ex vivo* BBB evaluating method, since our experimental paradigm did not require absolute quantification. Our results show that this fast and inexpensive new algorithm is specific enough to identify early BBB disruption, verified by the DHC-model [5]. It is, however, not absolutely quantitative but only suitable for relative measurements. Therefore, we suggest its use once there is need of evidence for whether BBB opened or not in a given region compared to the contralateral hemisphere. For an absolute quantification, we are in favor of the method described by Katayama and colleagues [13]. On the other hand, a clear advantage of our method over that of Katayama and colleagues is that the spatial information is better preserved where exactly in the tissue the BBB disruption did occur. Thus, our method is suitable in particular for the *ex vivo* assessment of BBB opening in cranial window experiments in comparison to sham controls. 

*In vivo* technology for the detection of BBB opening provides the advantage that dynamics of BBB opening can be studied. For this purpose, MR imaging is suitable as well as CT technology [14, 15]. Moreover, PET solutions have been discussed. For the specific purpose of cranial window experiments, an elegant method for the evaluation of BBB opening *in vivo* is fluorescent imaging of molecules with different molecular weights such as Lucifer Yellow using a CCD camera [16]. This method combines a high spatial with a high temporal resolution. Our algorithm can be used as a simple procedure to confirm the *in vivo* findings with another method *ex vivo*.

### 4.1. ET-1-Induced SDs as well as SDs Propagating in Naïve Tissue Do Not Lead to Significant Early BBB Opening

In 1993, Stanimirovic and colleagues reported that ET-1 increased cerebrovascular endothelium permeability in cultures derived from human brain capillary endothelial cells [17]. They suggested that this effect of ET-1 is due to receptor-specific activation of protein kinase C and intracellular calcium mobilization. Narushima and colleagues consequently showed in dogs that a single dose (40 pmol/animal) of ET-1 administered intracisternally led to enhanced fluorescein entry into the cerebrospinal fluid [18]. This effect was reversible by preadministration of the selective endothelin type A receptor (ET_A_) antagonist S-0139. Matsuo and colleagues showed that S-0139 administration decreased brain edema and albumin extravasation in a rat ischemia model, where the ischemia was not induced by ET-1 but due to middle cerebral artery filament occlusion [19]. This study suggested that ET-1 is released in the wake of focal ischemia in general and is involved via ET_A_ receptors in the delayed BBB disruption following focal ischemia. Cerebral ischemia and reperfusion injury seem to induce dynamic changes in the BBB permeability [20, 21]. Initial very slight changes are assumed to be reversible and less harmful. After a refractory period of more than three and less than five hours, a more marked and persistent BBB disruption develops which may interfere with therapies such as thrombolysis, shows side effects such as vasogenic edema, and is thus a target for therapeutic intervention [20, 21]. 

Our data suggest that there is no significant BBB disruption in the first few hours after topical administration of ET-1. The prolongation of SDs under ET-1 in contrast to SDs in naïve tissue indicated that the vasoconstrictor ET-1 produced conditions of an ischemic penumbra consistently with previous findings [6, 8]. Hence, it is very likely that we could have detected BBB disruption at a time point later than three hours, since focal ischemia is associated typically with BBB disruption in a delayed fashion after the third hour [22–24]. Thus, the lack of early BBB disruption in response to ET-1 provides in fact an argument that the BBB disruption induced by ET-1 in previous studies *in vivo* may have been secondary to ET-1-induced vasoconstriction and ischemia rather than due to a direct effect of ET-1 on the barrier. However, we cannot exclude that very subtle permeability changes escaped our BBB evaluating algorithm or that an increase in permeability occurs for smaller molecules than the albumin-Evans-blue complex. 

We neither found an early BBB opening after SDs propagating in naïve healthy tissue. This seems to contradict previous data in which matrix metalloproteinase (MMP)-9 was demonstrated to contribute to BBB permeability, edema formation, and vascular leakage after mechanically induced SDs in rats [4]. According to that study, MMP-9 activation occurred within the matrix of cortical blood vessels as early as 15 to 30 minutes and started within neurons more than three hours after SD induction. The authors reported an early BBB opening at three hours after SD induction, where BBB permeability was evaluated by quantification of vascular leakage of Evans blue dye using the method of Katayama and colleagues [13]. It is possible that our algorithm was not sensitive enough to detect a subtle early BBB opening. On the other hand, we used a less invasive method for SD induction (droplets of KCl at 150 mmol/L in a remote window) rather than a mechanical injury by pin prick. This might have influenced the time course of BBB opening.

## References

[1] Asahi M, Asahi K, Jung J-C, Del Zoppo GJ, Fini ME, Lo EH (2000). Role for matrix metalloproteinase 9 after focal cerebral ischemia: effects of gene knockout and enzyme inhibition with BB-94. *Journal of Cerebral Blood Flow and Metabolism*.

[2] Asahi M, Sumii T, Fini ME, Itohara S, Lo EH (2001). Matrix metalloproteinase 2 gene knockout has no effect on acute brain injury after focal ischemia. *NeuroReport*.

[3] Klohs J, Steinbrink J, Bourayou R (2009). Near-infrared fluorescence imaging with fluorescently labeled albumin: a novel method for non-invasive optical imaging of blood-brain barrier impairment after focal cerebral ischemia in mice. *Journal of Neuroscience Methods*.

[4] Gursoy-Ozdemir Y, Qiu J, Matsuoka N (2004). Cortical spreading depression activates and upregulates MMP-9. *Journal of Clinical Investigation*.

[5] Seiffert E, Dreier JP, Ivens S (2004). Lasting blood-brain barrier disruption induces epileptic focus in the rat somatosensory cortex. *Journal of Neuroscience*.

[6] Oliveira-Ferreira AI, Milakara D, Alam M (2010). Experimental and preliminary clinical evidence of an ischemic zone with prolonged negative DC shifts surrounded by a normally perfused tissue belt with persistent electrocorticographic depression. *Journal of Cerebral Blood Flow and Metabolism*.

[7] Dreier JP The role of spreading depression, spreading depolarization and spreading ischemia in neurological disease.

[8] Dreier JP, Kleeberg J, Alam M (2007). Endothelin-1-induced spreading depression in rats is associated with a microarea of selective neuronal necrosis. *Experimental Biology and Medicine*.

[9] Lindauer U, Villringer A, Dirnagl U (1993). Characterization of CBF response to somatosensory stimulation: model and influence of anesthetics. *American Journal of Physiology*.

[10] Dreier JP, Kleeberg J, Petzold G (2002). Endothelin-1 potently induces Leão’s cortical spreading depression in vivo in the rat: a model for an endothelial trigger of migrainous aura?. *Brain*.

[11] Jorks D, Major S, Oliveira-Ferreira AI, Kleeberg J, Dreier JP (2011). Endothelin-1(1-31) induces spreading depolarization in rats. *Acta Neurochirurgica Supplement*.

[12] Ehrlich P (1885). *Das Sauerstoff-Bedürfnis des Organismus Eine Farbenanalytische Studie*.

[13] Katayama S, Shionoya H, Ohtake S (1978). A new method for extraction of extravasated dye in the skin and the influence of fasting stress on passive cutaneous anaphylaxis in guinea pigs and rats. *Microbiology and Immunology*.

[14] Tomkins O, Friedman O, Ivens S (2007). Blood-brain barrier disruption results in delayed functional and structural alterations in the rat neocortex. *Neurobiology of Disease*.

[15] Hjort N, Wu O, Ashkanian M (2008). MRI detection of early blood-brain barrier disruption: parenchymal enhancement predicts focal hemorrhagic transformation after thrombolysis. *Stroke*.

[16] Prager O, Chassidim Y, Klein C, Levi H, Shelef I, Friedman A (2010). Dynamic in vivo imaging of cerebral blood flow and blood-brain barrier permeability. *NeuroImage*.

[17] Stanimirovic DB, McCarron R, Bertrand N, Spaiz M (1993). Endothelins release Cr from cultured human cerebromicrovascular endothelium. *Biochemical and Biophysical Research Communications*.

[18] Narushima I, Kita T, Kubo K (1999). Contribution of endothelin-1 to disruption of blood-brain barrier permeability in dogs. *Naunyn-Schmiedeberg’s Archives of Pharmacology*.

[19] Matsuo Y, Mihara SI, Ninomiya M, Fujimoto M (2001). Protective effect of endothelin type A receptor antagonist on brain edema and injury after transient middle cerebral artery occlusion in rats. *Stroke*.

[20] Jin AY, Tuor UI, Rushforth D (2010). Reduced blood brain barrier breakdown in P-selectin deficient mice following transient ischemic stroke: a future therapeutic target for treatment of stroke. *BMC Neuroscience*.

[21] Jin R, Yang G, Li G (2010). Molecular insights and therapeutic targets for blood-brain barrier disruption in ischemic stroke: critical role of matrix metalloproteinases and tissue-type plasminogen activator. *Neurobiology of Disease*.

[22] Kuroiwa T, Ting P, Martinez H, Klatzo I (1985). The biphasic opening of the blood-brain barrier to proteins following temporary middle cerebral artery occlusion. *Acta Neuropathologica*.

[23] Pluta R, Lossinsky AS, Wisnieuwski HM, Mossakowski MJ (1994). Early blood-brain barrier changes in the rat following transient complete cerebral ischemia induced by cardiac arrest. *Brain Research*.

[24] Belayev L, Busto R, Zhao W, Ginsberg MD (1996). Quantitative evaluation of blood-brain barrier permeability following middle cerebral artery occlusion in rats. *Brain Research*.

